# Effect of modified Shenqi pill combined with conventional western medicine on diabetic nephropathy and its influence on netrin-1 level and renal function

**DOI:** 10.4314/ahs.v25i1.31

**Published:** 2025-03

**Authors:** Xiaoli Zhang, Cheng'e Gao, Xiaoshuang Yin

**Affiliations:** 1 Department of Endocrinology, Affiliated Hospital of Shandong University of traditional Chinese Medicine, Jinan, China; 2 Outpatient Service Center, Affiliated Hospital of Shandong University of traditional Chinese Medicine, Jinan, China; 3 Department of Nephrology, Affiliated Hospital of Shandong University of traditional Chinese Medicine, Jinan, China

**Keywords:** Diabetic nephropathy, modified Shenqi pill, western medicine, combined therapy, nerve guidance factor-1, renal function

## Abstract

**Background:**

Our aim in this study was to investigate the efficacy of modified Shenqi pills combined with conventional western medicine in patients with diabetic nephropathy.

**Methodology:**

150 patients with diabetic nephropathy were divided into a western medicine group (50 cases, conventional western medicine), a traditional Chinese medicine group (50 cases, modified Shenqi pills), and an observation group (50 cases, modified Shenqi pills combined with conventional western medicine). The values of netrin-1, 24-hour urinary protein, blood urea nitrogen (BUN), creatinine (Scr), symptom score, and clinical efficacy were compared among three groups before and after treatment.

**Results:**

The netrin-1, 24h urine protein, BUN and Scr values, the total score of main symptoms, and total score of secondary symptoms in the observation group were lower than those in the traditional Chinese medicine group and western medicine group at 3 months after treatment, and the difference was statistically significant. The total effective rate in the observation group (100.00%) was significantly higher than that in the traditional Chinese medicine group (86.00%) and western medicine group (78.00%), and the difference was statistically significant.

**Conclusion:**

Combination of modified Shenqi Pills with conventional western medicine can achieve a more satisfactory effect in diabetic nephropathy, by reducing Netrin-1, promoting renal function recovery, eliminating edema and decreasing blood pressure.

## Introduction

Diabetic nephropathy is a significant complication of diabetes and is characterized by symptoms such as microalbuminuria, edema, hypertension, palpitations, dizziness, diarrhea, nausea, and polyuria. If left untreated, it can lead to end-stage kidney disease, resulting in disturbances in water and electrolyte balance, and triggering a series of illnesses that can threaten the patient's life and health. Therefore, diabetic patients need to pay attention to the prevention and treatment of diabetic nephropathy. Patients with suspected symptoms should be admitted to the hospital in a timely manner^1^,^2^.

Western medicine is currently the main treatment for diabetic nephropathy. Lowering blood glucose and blood pressure can effectively reduce hyperglycemia toxicity and delay the progression of renal function, resulting in significant disease control. However, long-term western medicine treatment may increase the toxic side effects of drugs and affect medication efficacy in patients. Therefore, there is a need to explore other treatment options for diabetic nephropathy^3^,^4^.

Modified Shenqi Pills have been shown to be effective in improving water and detumescence, warming the kidneys, and dissipating qi, and can relieve symptoms of kidney disease such as edema, water, and electrolyte imbalance. However, there are few studies that have examined the combination of modified Shenqi pills and conventional western medicine in diabetic nephropathy. Therefore, this study aims to analyze the clinical efficacy of conventional Western medicine-modified Shenqi pills and modified Shenqi pills + conventional Western medicine in patients with diabetic nephropathy (yin and yang deficiency evidence) by comparing and analyzing the effect of this combined Chinese and Western medicine treatment on the expression level of neurotransmitter-1 (Netrin-1) and renal function indexes^5^,^6^.

Therefore, there is a need to explore other treatment options, such as the combination of modified Shenqi pills and conventional western medicine. This study aims to analyze the clinical efficacy of this combined treatment on the expression level of neurotransmitter-1 (Netrin-1) and renal function indexes, which could provide a reference for clinical treatment of diabetic nephropathy. Diabetic nephropathy is one of the most important microvascular complications of diabetes, and its common clinical manifestations are microalbuminuria, edema, hypertension, palpitations, dizziness, etc. Some patients may present with diarrhea, nausea, and polyuria symptoms. Without timely treatment, the condition can progress to end-stage kidney disease (renal failure), leading to disturbances in water and electrolyte balance, and triggering a series of illnesses that pose a serious threat to the patient's life and health^1^,^2^. Therefore, diabetic patients need to pay attention to the prevention and treatment of diabetic nephropathy. Patients with edema, hypertension and other suspected symptoms should be admitted to the hospital in a timely manner. At present, diabetic nephropathy is mainly treated with western medicine. Generally, a significant disease control effect can be achieved by lowering blood glucose and lowering blood pressure, which can effectively reduce hyperglycemia toxicity and delay the progression of renal function^3^,^4^. However, long-term western medicine treatment may increase the toxic side effects of drugs and affect the efficacy of medication in patients. The modified Shenqi Pills have the effects of improving water and detumescence, warming the kidneys, and dissipating qi, and can effectively relieve symptoms of kidney disease such as edema, water, and electrolyte imbalance^5^,^6^. Currently, most domestic studies are focused on western medicine's application to diabetic nephropathy, or on traditional Chinese medicine's application to diabetic nephropathy. Few studies have examined the combination of modified Shenqi pills and conventional western medicine in diabetic nephropathy. Therefore, in this study, patients with diabetic nephropathy (yin and yang deficiency evidence) were targeted for intervention.

The clinical efficacy of conventional Western medicine-modified Shenqi pills and modified Shenqi pills + conventional Western medicine was compared and analyzed. The aim was to analyze the effect of this combined Chinese and Western medicine treatment on the expression level of neurotransmitter-1 (Netrin-1) and renal function indexes, in order to provide a reference for the choice of clinical treatment for diabetic nephropathy.

## Patients and methods

### Patients

From January 2020 to December 2021, 150 patients with diabetic nephropathy treated at our hospital were selected as the research subjects. Inclusion criteria: (1) patients who met the Western medical diagnostic and treatment criteria for diabetic nephropathy in the “Expert Consensus on Multidisciplinary Diagnosis and Treatment of Diabetic Nephropathy”^7^, with symptoms such as edema and hypertension, and renal biopsy indicating diabetic nephropathy; (2) patients who met the Chinese medical diagnostic and treatment criteria for diabetic nephropathy (yin-yang deficiency syndrome) in the “Guidelines for the Prevention and Treatment of Diabetic Nephropathy in Traditional Chinese Medicine”^8^, with the main symptoms of fatigue, palpitations, shortness of breath, dizziness, tinnitus, spontaneous sweating, and night sweats, and secondary symptoms of thirst, irritability, insomnia, premature ejaculation, and a tongue and pulse image of pale red tongue, scanty or moss-free coating, and slippery or weak pulse; (3) patients aged ≥18 years; (4) patients with complete general data; and (5) patients classified according to the degree of glomerular injury as Grade I-III. Exclusion criteria: (1) severe allergic reactions to medications used in this study; (2) previous treatment for diabetic nephropathy before enrollment; (3) concomitant kidney diseases such as glomerulonephritis and chronic pyelonephritis; (4) pregnant or lactating women; (5) concomitant other diabetic complications; (6) tumors or hematologic disorders; (7) recent rapid deterioration of renal function; (8) recent infectious or contagious diseases before enrollment; (9) use of glucocorticoids, immunosuppressants, probiotics, or other therapies affecting Netrin-1 levels and renal function within 2 months before enrollment; (10) psychiatric disorders or impaired compliance; (11) participation in other experimental studies or withdrawal from this study.

Participants were evenly divided into three groups using random number tables: the western medicine group (50 cases, treated with conventional western medicine), the traditional Chinese medicine group (50 cases, treated with modified Shenqi pills), and the observation group (50 cases, treated with modified Shenqi pills combined with conventional western medicine). There was no significant difference in the general data between the two groups (P > 0.05), as shown in Table 1. The patient and family agreed and signed the informed consent form, which was reviewed and approved by the Affiliated Hospital of Shandong University of Traditional Chinese Medicine ethics committee.

**Table 1:** The general data between the two groups

Group	n	Male(%)	Age(years)	duration of diabetes(years)	duration of diabetic nephropathy(months)
western medicine group	50	26(52%)	56.05 ± 6.89	6.68 ± 1.81	17.06 ± 3.24
traditional Chinese medicine group	50	28(56%)	55.58 ± 6.53	7.51 ± 1.92	16.08 ± 3.14
observation group	50	23(46%)	55.53 ± 6.72	6.81 ± 1.86	17.51 ± 3.27
F/*χ*^2^		1.014	0.091	2.868	2.583
P		0.602	0.913	0.060	0.079

## Methods

### Treatment methods

In the western medicine group, conventional western medicine was used to treat the patients. Personalized western medicine treatment was developed according to the patient's condition, and the common drugs were losartan potassium tablets (Merck Sharp & Dohme Limited, UK, GYZZ J20180054, strength 50 mg × 7 tablets/box), orally, 50 mg/time, once a day; metformin hydrochloride tablets (Sino-American Shanghai Squibb Pharmaceutical Co., Ltd., Shanghai, China, GYZZ H20023370, strength 0.5 g × 20 tablets/box), orally, with an initial medication of 0.5 g/time, twice a day. After 1 week of medication, the dose could be appropriately increased to 1.0 g/time, twice a day. Acarbose tablets (Hangzhou Sino-American East Pharmaceutical Co., Ltd., GYZZ H20020202, strength 50 mg × 60 tablets/box), orally (immediately before meals or chewing with the previous mouth foods), with an initial medication of 50 mg/time, third a day.

As the patient's condition improves after a week of medication, the dose may be appropriately increased to 100 mg/time, three times a day, with a maximum dose of 600 mg/day. Repaglinide Tablets (Boehringer Ingelheim Pharma GmbH & Co.KG, Registration No. H20171153, strength 1 mg × 30 tablets/box), orally (taken 15 min before meals), with the initial dose of 0.5 mg/time, third a day. After 1 week of medication, the dose can be appropriately increased to 1 mg/time, third a day, with a maximum dose of 16 mg/d according to the patient's condition. Captopril Tablets (Guangdong Petty Pharmaceutical Co., Ltd., GYZZ H44021595, strength 25 mg × 100 tablets/bottle), orally, 12.5 mg/time, third a day. According to the patient's condition, the dose can be increased appropriately to 25-50 mg/time, third a day, and the maximum dose is 150 mg/d. Medication was administered for 3 months.

Chinese medicine group: treated with modified Shenqi pills. Modified Shenqi Wan formula is 15 g each of Rehmannia glutinosa, Yam, Poria cocos, Plantago asiatica, Aconite root, Paeonia lactiflora, and Achyranthes bidentata, 12 g each of Cornus officinalis and Alisma orientalis, and 10 g of Guangui. Add 400 mL water to boil to 300 mL, divided into two warm doses in the morning and evening (150 mL/time), continuous medication for 3 months.

### Observation group

Treated with modified Shenqi pills combined with conventional western medicine. The modified Shenqi pills in this group were completely consistent with the traditional Chinese medicine group. The conventional western medicine in this group was consistent with the western medicine group combined drug treatment for 3 months.

### Equipment and Reagents

Centrifuge (Model No. LXJ-II) was purchased from Shanghai Medical Analytical Instrument Factory. The cryopreservation refrigerator was purchased from Wuhan Hans Instrument & Equipment Co., Ltd. (Wuhan, China). The thermostatic incubator (Model No. DNP-9082) was purchased from Ningbo Jiangnan Instrument Factory (Ningbo, China). An automatic biochemical analyzer (Model No. BK-200) was purchased from Boke Holding Group Co., Ltd. (Jinan, China). Netrin-1 enzyme-linked immunosorbent assay kit and supporting reagents were purchased from Shanghai enzyme-linked Biotech Co., Ltd. (Shanghai, China)

### Test method

Three milliliters of fasting cubital venous blood was collected from the patients in the morning, centrifuged (10 min, 15 cm × 3000 r/min), and serum was separated and stored at low temperature until testing, and removed 30 min before testing and placed at room temperature. Serum netrin-1 expression levels were measured with enzyme-linked immunosorbent assay kits and matching reagents. The 24-hour urinary protein, blood urea nitrogen (BUN), and creatinine (serum creatinine Scr) were detected by an automatic biochemical analyzer.

### Observation indicates

(1) Netrin-1 levels were compared among the three groups at admission and 3 months after treatment. (2) The renal function indexes of the three groups were compared before and after treatment, including 24 h urinary protein, BUN, and Scr at admission and 3 months after treatment. (3) Compare the symptom scores of the three groups before and after treatment, refer to the Guidelines for Clinical Research of New Drugs of Traditional Chinese Medicine in the Treatment of Diabetes9 TCM symptom scores, and score the main symptoms (fatigue, palpitation, shortness of breath, dizziness, tinnitus, spontaneous sweating, and night sweats) and secondary symptoms (thirst like to drink, upset insomnia, spermatorrhea, and premature ejaculation), each symptom score is 0-3 points, the total score of the main symptoms is 0-12 points, and the total score of the secondary symptoms is 0-9 points, the higher the score, the more severe the symptoms. (4) The clinical efficacy was compared, and the therapeutic effect that could be achieved from the start of drug treatment to 3 months after treatment was statistically analyzed, which was significantly netrin-1 < 300 ng/mL, Scr < 90 µmol/L, the total score of main symptoms < 2 points, total score of secondary symptoms < 1 point, edema, hypertension, and other symptoms subsided; effective was 300 ng/mL ≤ netrin-1 < 350 ng/mL, 90 µmol/L ≤ Scr < 110 µmol/L, total score of main symptoms < 4 points, total score of 1 point ≤ total score of secondary symptoms < 2 points, edema, hypertension, and other symptoms were significantly improved; ineffective was netrin-1 ≥ 350 ng/mL, Scr ≥ 110 µmol/L, the total score of main symptoms ≥ 4 points, the total score of secondary symptoms ≥ 2 points, edema, hypertension, and other symptoms were not significantly improved. Overall response rate = significant rate + response rate.

### Statistical analysis

Statistical analysis was performed using Statistical Product and Service Solutions (SPSS) 23.0 software (IBM, Armonk, NY, USA). Measurement data were presented as a normal distribution (x±s), of two independent samples we used the t-test for independent samples, while the test for three independent samples used the ANOVA; enumeration data were presented as rate (%), x2 test was used for intergroup comparison, and P < 0.05 indicated a statistically significant difference.

## Results

### Compare netrin-1 values before and after treatment in the three groups

There was no significant difference in netrin-1 values between the three groups at admission (P > 0.05). The netrin-1 detection value 3 months after treatment in the observation group was significantly lower than that in the traditional Chinese medicine group and western medicine group, and the difference was statistically significant (P < 0.05). See Table 2.

**Table 2:** Comparison of netrin-1 values before and after treatment in the three groups (x±s, ng/mL)

Group	Number of cases	Admission	3 months after treatment
Observation group	50	378.65 ± 27.65	240.58 ± 20.13
TCM group	50	379.34 ± 28.04	321.45 ± 22.54
Western medicine group	50	371.65 ± 27.57	344.08 ± 23.78
F value	-	1.180	300.330
P value	-	0.312	< 0.001

### Compare renal function indicators before and after treatment in the three groups

There was no significant difference in 24 h urinary protein, BUN and Scr among the three groups at admission (P > 0.05). The 24-hour urinary protein, BUN and Scr in the observation group were lower than those in the traditional Chinese medicine group and western medicine group at 3 months after treatment, and the differences were statistically significant (P < 0.05). See Table 3.

**Table 3:** Comparison of renal function indicators before and after treatment in the three groups [x±s]

Group	Number of cases	24 h urine protein (mg)		BUN (mmol/L)		Scr (µmol/L)	
		Admission	3 months after treatment	Admission	3 months after treatment	Admission	3 months after treatment
Observation group	50	2.44 ± 0.16	1.12 ± 0.12	11.32 ± 2.15	7.07 ± 0.84	144.83 ± 12.21	88.45 ± 7.46
TCM group	50	2.38 ± 0.12	1.67 ± 0.14	11.27 ± 2.11	8.96 ± 0.95	145.01 ± 12.54	98.56 ± 8.86
Western medicine group	50	2.41 ± 0.14	1.85 ± 0.16	11.41 ± 2.18	9.43 ± 1.01	144.15 ± 12.35	117.63 ± 9.12
F value	-	2.270	364.0 10	0.050	89.06 0	0.070	151.5 40
P value	-	0.107	<0.001	0.947	<0.001	0.935	<0.001

### Compare the symptom scores of the three groups before and after treatment

There was no significant difference in the total score of main symptoms and secondary symptoms among the three groups at admission (P > 0.05). The total score of main symptoms and secondary symptoms in the observation group 3 months after treatment were lower than those in the traditional Chinese medicine group and western medicine group, and the differences were statistically significant (P < 0.05). See Table 4.

**Table 4:** Comparison of symptom scores before and after treatment among the three groups (x±s, points)

Group	Number of cases	Total score of main symptoms		Total secondary symptom score	
		Admission	3 months after treatment	Admission	3 months after treatment
Observation group	50	8.12 ± 1.25	1.97 ± 0.14	2.55 ± 0.32	0.86 ± 0.11
TCM group	50	8.02 ± 1.18	3.06 ± 0.88	2.58 ± 0.35	1.09 ± 0.16
Western medicine group	50	8.07 ± 1.22	3.87 ± 0.93	2.49 ± 0.29	1.78 ± 0.21
F value	-	0.080	82.200	1.020	920.300
P value	-	0.919	< 0.001	0.363	< 0.001

### Compare the clinical efficacy of the three groups

The total effective rate in the observation group (100.00%) was significantly higher than that in the traditional Chinese medicine group (86.00%) and western medicine group (78.00%), and the difference was statistically significant (P < 0.05). See Table 5.

**Table 5:** Comparison of clinical efficacy among three groups [cases (%)]

Group	Number of cases	Significant	Effective	Invalid	Overall response rate
Observation group	50	24 (48.00)	26 (52.00)	0 (0)	50 (100.00)
TCM group	50	14 (28.00)	29 (56.00)	7 (14.00)	43 (86.00)
Western medicine group	50	11 (22.00)	28 (56.00)	11 (22.00)	39 (78.00)
X^2^ value	-	8.426	0.378	11.742	11.742
P value	-	0.015	0.828	0.003	0.003

## Discussion

### Combination therapy reduces netrin-1 expression

Diabetic nephropathy is a long-term complication of diabetes that is characterized by proteinuria and progressive decline in the glomerular filtration rate^10^-^12^. Western medicine attributes the pathology to abnormal glucose metabolism, which causes hyperglycemia that worsens the glucose load of the kidney, leading to renal impairment and nephropathy^13^-^15^. In addition, other factors such as oxidative stress, immune inflammation, renal hemodynamic changes, and genetics also increase the risk of diabetic nephropathy. Traditional Chinese medicine (TCM) considers diabetic nephropathy as a manifestation of “Xiaoke” or “Kidney Xiaoke” that results from Qi and Yin deficiency, which ultimately leads to the accumulation of water and dampness, causing symptoms such as edema and hypertension. This study examined the clinical efficacy of modified Shenqi pills combined with conventional western medicine treatment for diabetic nephropathy.

The results inicated that the combined treatment reduced the netrin-1 expression level, which is a protein associated with the apoptosis of endothelial cells, inflammation, and angiogenesis. The modified Shenqi pill, which contains various herbs, has the effects of warming kidney and dissipating qi, benefiting water and detumescence, and tonifying fire and assisting yang, which help alleviate symptoms of edema and hypertension^16^-^18^. Diabetic nephropathy mainly refers to long-term diabetes caused by proteinuria and the glomerular filtration rate showed a progressive decline.

In western medicine, abnormal glucose metabolism is believed to be a contributing factor to diabetic nephropathy. The hyperglycemic state caused by diabetes will make about 50% of glucose metabolized in the kidney, which aggravates the glucose load of the kidney while reducing the risk of complications such as ketoacidosis and hyperosmolar coma, and then induces the occurrence of renal impairment and nephropathy^10^. In addition, oxidative stress, immune inflammation, renal hemodynamic changes, genetics, and other factors have also been shown to increase the risk of diabetic nephropathy^11^. In terms of traditional Chinese medicine diagnosis and treatment, diabetic nephropathy is often categorized as “Xiaoke” (diabetes), “Kidney Xiaoke” (kidney diabetes), or within the diagnostic category of “edema” and “turbid urine” that are secondary to Xiaoke disease. It is believed that the main pathogenesis of diabetic nephropathy is Qi and Yin deficiency. Prolonged illness entering the Luo (a network of collaterals) affects the kidneys, which are the site where the collaterals converge. Failure to cure Xiaoke disease over a long period can injure Yin and consume Qi, leading to phlegm-heat stagnation and blood stasis that obstruct the collaterals, thereby damaging Yin and Yang, impairing the spleen and kidneys, and causing water and dampness to accumulate an-flood the skin, ultimately leading to the onset of symptoms such as edema and hypertension^12^.

To improve the clinical efficacy of diabetic nephropathy, modified Shenqi pills combined with conventional western medicine treatment were applied to the diagnosis and treatment of patients with such diseases in this study. The study data showed that the netrin-1 detection values 3 months after treatment in the observation group were significantly lower than those in the traditional Chinese medicine group and western medicine group (P < 0.05), suggesting that the combined treatment could reduce the netrin-1 expression level. The reasons are as follows: With the deepening of related research, some scholars have successively proposed “cell death mechanism”, “exosome”, “epigenetics” and “pyroptosis theory” in recent year-stoo explain the specific pathology leading to diabetic nephropathy. Among them, the mechanism of cell death is a hotly debated topic in such theories, and it has been shown that factors such as glucose metabolism disorders and a variety of cytokines are related to the development of diabetic nephropathy, while apoptosis is closely related to the development of such factors^13^. Song Qimeng^14^ and other studies showed that high glucose caused human glomerular endothelial cell injury and promoted human glomerular endothelial cell apoptosis. If diabetic patients are in a state of hyperglycemia for a long time, it may increase the rate of endothelial cell apoptosis, leading to a large number of oxygen free radicals released, aggravate the inflammatory response, aggravate the degree of renal impairment, and then mediate the occurrence of nephropathy. Netrin-1 is a highly conserved secreted protein that is mainly derived from midline glial cells in the developing spinal cord. Under normal physiological conditions, netin-1 promotes endothelial cell migration, proliferation, and angiogenesis, and reduces tissue fibrosis^15^. Studies by He Jinhua et al.^16^ showed a positive correlation between type 2 diabetic nephropathy and urinary netrin-1 expression levels. These results suggest that the more severe the diabetic nephropathy, the higher the netrin-1 detection value of patients, and conversely if the netrin-1 detection value shows a downward trend, it indicates that diabetic nephropathy patients may tend to improve their condition on the side. This may be because netrin-1 is involved in the process of cell, angiogenesis, apoptosis, etc. While the apoptosis rate of endothelial cells increases under a high glucose state, the expression level of netrin-1 is on the rise to antagonize diabetes-induced endothelial cell apoptosis, inhibit inflammation, promote the proliferation and repair of endothelial cells, and achieve self-protection of the body. In this study, after treatment with western medicine, the values of netrin-1 in patients with diabetic nephropathy decreased, that is, western medicine treatment can achieve a certain effect of glucose control and antihypertensive, to control the development of nephropathy. In the treatment of the modified Shenqi pill, Rehmannia glutinosa has the effects of tonifying blood and nourishing yin, benefiting essence and filling marrow, which is the monarch drug; Yam and Poria cocos have the effects of invigorating spleen and tonifying lung, solidifying kidney and benefiting essence, benefiting spleen and nourishing heart, and light infiltration and removing dampness, which is the minister drug; Plantago asiatica, Paeonia lactiflora, Achyranthes bidentate, and Cornus officinalis have the effects of nourishing water and dredging, permeating dampness and antidiarrheal, clearing away heat and phlegm, promoting blood circulation and removing blood stasis, tonifying liver and kidney, and strengthening muscles and bones, which is the adjuvant drug; Combined use of various drugs has the effects of warming kidney and dissipating qi, benefiting water and detumescence, tonifying fire and assisting yang, and can effectively improve edema, hypertension, and other symptoms. At the same time, it has been mentioned that the combination of Rehmannia glutinosa, Cornus officinalis, and Yam has the ability to nourish yin and unifying essence, giving consideration to both source and flow, and helping seal and store, and then with the regulation and assistance of aconite root warming yang, dissipating qi and benefiting water, it can lead to drug force directly driving down, further improve its overall efficacy, achieve complementary yin and yang, promote the recovery of renal function, and reduce renal injury^18^.

Therefore, after the observation group was treated with modified Shenqi Pills combined with conventional western medicine, while lowering blood glucose, lowering blood pressure, and improving the high glucose status of the body, modified Shenqi Pills could promote the recovery of renal function and improve nephropathy. Correspondingly, with the improvement of the patient's nephropathy, its netin-1 detection value showed a downward trend, indicating that endothelial cell apoptosis was controlled to some extent, benign circulation was easily formed, and endothelial cell proliferation and differentiation and tissue repair were promoted.

### Combined treatment can improve renal function parameters

The data of this study showed that the 24-hour urinary protein, BUN, and Scr in the observation group were lower than those in the traditional Chinese medicine group and western medicine group 3 months after treatment (P < 0.05), suggesting that the combined treatment could promote the recovery of renal function. The reasons for analysis are as follows: Several studies have shown that acarbose tablets, repaglinide tablets, and other western medicine drugs can achieve a more significant hypoglycemic and glucose-control related, the improvement of high glucose status is conducive to the control and recovery of kidney disease and can achieve a more significant medication effect in diabetic nephropathy^19^,^20^. At the same time, with the promotion of TCM medicine and the deepening of related research, in modern pathology, Rehmannia glutinosa extracts a variety of chemicals such as flavin, difloridine, amino acids, and catalpol, which have the effects of delaying aging, improving inflammation, and tonifying blood, and can effectively regulate the symptoms of yin deficiency^21^. Yam has hypoglycemic and lipid-lowering effects and immunoregulatory effects in modern pharmacology; Poria cocos, Plantago asiatica, Paeonia lactiflora, and Achyranthes bidentata have diuretic and hepatoprotective effects; Aconite root has vasodilator and blood circulation improving effects; Cornus officinalis has antihypertensive, antioxidant, and anti-aging effects; Alisma orientalis has hypolipidemic and diuretic effects; and Guangui has antihypertensive, cooling, and sedative effects.

Combined use of various drugs can play a role in hypoglycemic antihypertensive lipid-lowering, improving inflammatory response, promoting hemodynamic recovery, diuresis, and eliminating edema.

Therefore, compared with single western medicine and traditional Chinese medicine treatment, the combination can achieve a more significant recovery of renal function and further reduce 24 h urinary protein, BUN, and Scr test values.

### Combined therapy can reduce symptom scores and improve clinical efficacy

The data of this study showed that the total score of main symptoms and secondary symptoms in the observation group were lower than those in the TCM group and western medicine group 3 months after treatment, and the total effective rate in the observation group was higher than that in the TCM group and western medicine group (P < 0.05), suggesting that the combined treatment could reduce the symptom score and improve the clinical efficacy.

The reasons for analysis are as follows: Studies have shown that substances similar to sitosterol are one of the core active components of the main medicinal materials of Shenqi Pills, which have a positive effect on improving cell proliferation and differentiation, and have the effect of lowering blood glucose and have certain application value in diabetic nephropathy^22^. In addition, similar sitosterol components have immunomodulatory effects, reduce oxidative stress, improve inflammatory response, etc., which are conducive to alleviating the inflammatory response caused by renal injury and play an important role in promoting the recovery of renal function. Therefore, based on conventional western medicine treatment combined with modified Shenqi pills, while lowering blood glucose and controlling blood pressure, and improving the state of a high glucose environment, modified Shenqi pills can play a role in promoting endothelial cell proliferation and differentiation, promoting tissue repair, improving renal injury, promoting the regression of edema and microalbuminuria symptoms, and play an important role in improving its overall medication effect.

## Limitations

Firstly, the sample size selected for the study was relatively small, which may have led to some errors in the results.

Additionally, the lack of long-term follow-up of patients means that the efficacy and potential toxic side effects of using modified Shenqi pills in combination with conventional Western medicine over an extended period still require further investigation. Therefore, while the initial results are promising, further clinical exploration is needed to confirm the broad efficacy of this approach. However, as far as this study is concerned, there are still the following shortcomings: 1. the number of study samples selected is small, and there are some errors in the study results; 2. no long-term follow-up of patients failed to confirm the toxic side effects and efficacy of modified Shenqi pills + conventional western medicine after long-term treatment, so whether the study results have broad efficacy still needs to be confirmed by further clinical exploration.

## Conclusion

In summary, the application of Jiawei Shenqi Pills combined with conventional western medicine in the diagnosis and treatment of diabetic nephropathy can reduce the expression level of netrin-1, promote the recovery of renal function indicators, improve the symptoms of diabetic nephropathy, and improve the clinical efficacy, which is worthy of clinical application.

## Data Availability

The datasets used and analyzed during the current study are available from the corresponding author upon reasonable request.
